# Influence of isolated coronary artery bypass graft on moderate functional mitral regurgitation

**DOI:** 10.34172/jcvtr.025.33277

**Published:** 2025-06-28

**Authors:** Hakimeh Sadeghian, Babak Oloomi, Seyed Hossein Ahmadi Tafti, Akbar Shafiee, Arash Jalali, Mohammad Amin Masoumi

**Affiliations:** ^1^Department of Cardiology, Dr. Shariati Hospital, Tehran University of Medical Science, Tehran, Iran; ^2^Tehran Heart Center, Cardiovascular Diseases Research Institute, Tehran University of Medical Science, Tehran, Iran; ^3^Abarkuh khatam al-anbia Hospital, Shahid Sadoughi University of Medical Sciences, Abarkuh, Iran

**Keywords:** Mitral valve, Functional moderate mitral regurgitation, Coronary artery bypass graft

## Abstract

**Introduction:**

The natural course and clinical significance of moderate mitral regurgitation (MR) in patients undergoing isolated coronary artery bypass graft (CABG) surgery are still debated. This study aimed to determine the course of moderate functional MR after CABG.

**Methods:**

In this registry-based cohort, patients who underwent isolated elective CABG at Tehran Heart Center between 2010 and 2017 were included. Transthoracic echocardiography was performed at baseline before CABG and after 12 months of follow-up. The outcomes of interest were both improvement and progression of MR during the study.

**Results:**

Among 291 patients with moderate functional MR, the mean age was 66.1±9.6 years, and 204 (70.1%) were males. Most of the study population had extensive coronary disease (240 patients; 82.5% with thee-vessel disease). Moreover, 101 patients (34.7%) had suffered a prior myocardial infarction. The mean LVEF before CABG was 42.0±9.9 and 145 patients (49.8%) had an LVEF≤40% prior to surgery.The median follow-up duration was 10.1 months (9.0-11.2). Only four patients had improvements in MR at follow-up. Eleven patients (3.8%) in the study population showed worsening MR after isolated CABG. In this group of patients, mean LVEF dropped from 44.1±10.9 at baseline to 41.8±11.5 during follow-up Due to the low number of cases with regression and progression, an analysis of predictors of MR change was not performed.

**Conclusion:**

This study showed that in patients with moderate functional MR, isolated CABG did not result in significant changes in the degree of MR.

## Introduction

 Mitral regurgitation (MR) is a common condition among patients with coronary artery disease (CAD), and among patients undergoing coronary artery bypass grafting (CABG) surgery, 3-7% demonstrate varying degrees of MR.^1,2^ MR in patients with CAD is associated with a higher risk of mortality and a higher incidence of heart failure.^3,4^ Ischemic MR (IMR) is caused by LV remodeling, papillary muscle displacement, and annular dilation.^5^ Thus, it is expected that revascularization and relieving the ischemic injury would positively affect mitral valve function.

 For patients with severe functional MR undergoing CABG, evidence suggests that concomitant mitral valve surgery is associated with better outcomes. The previous 2014 guidelines suggested mitral valve surgery when CABG is undertaken for moderate functional MR (class IIb, with a level of evidence of C), but in the latest guidelines, this is not recommended for moderate functional mitral regurgitation.^6,7^ However, the clinical significance of lower degrees of MR at the time of CABG is unclear. The current evidence about the natural course of moderate MR in patients undergoing CABG is conflicting and inconclusive.^3,8,9^ Besides, current guidelines fail to give recommendations on the benefit of CABG alone in improving MR. Thus, it would be critical to identify predictors of severe MR after CABG to select patients who would require mitral valve surgery prospectively.

 In this context and based on the findings in our previous study on percutaneous coronary intervention candidates,^10^ we aimed to investigate the clinical course of MR in patients with moderate functional MR who underwent CABG and to determine the clinical associations of regression and progression of MR in this population.

## Materials and Methods

###  Study design and population

 In this registry-based cohort study, data of all patients who underwent isolated and elective CABG at Tehran Heart Center between 2010 and 2017 were retrieved and analyzed from the registry of our center. Candidates for isolated CABG, aged 20 years or older, who had chronic moderate functional MR in their preoperative transthoracic echocardiography were considered eligible. The exclusion criteria were a history of previous valvular surgery, CABG, angioplasty, arrhythmia ablation, or implantation of a cardiac pacing device, presence of severe valvular disease, and a diagnosis of rheumatic MR or mitral valve prolapse.

###  Definitions and data collection

 Diagnosis of MR and determination of its degree was based on preoperative transthoracic echocardiography (TTE). The definition of mild, moderate, and severe functional MR was based on the recommendations of the American Society of Echocardiography.^7,11^ According to the color flow jet area of MR, an area < 4 cm^2^ or < 20% of the left atrium (LA) area or vena contracta < 3mm was considered mild MR. Severe MR was defined as a regurgitant jet area > 10 cm^2^ or > 40% of the LA area or vena contracta ≥ 7mm. Moderate MR was approved upon the presence of a regurgitant jet area on color flow Doppler that was larger than mild and smaller than the size to be considered severe.^10^

 CABG surgeries were performed by expert cardiac surgery teams using standard conventional methods. Generally, after prep and drape under general anesthesia, a mid-sternotomy was performed before harvesting LIMA and/or SVG for the anastomosis. All the data for this study were retrieved from the Cardiac Surgery databank of Tehran Heart Center.^12,13^ Demographic data, clinical characteristics, and surgical features, including age, sex, cardiovascular risk factors, comorbidities, drug history, results of coronary angiography, use of cardiopulmonary pump during surgery, and the number of coronary grafts were obtained from databank. Moreover, heart rate and cardiac rhythm (sinus rhythm versus atrial fibrillation) were determined based on preoperative electrocardiogram and were recorded in the database. Data of TTE findings pre- and post-CABG were obtained from the Tehran Heart Center echocardiography databank and included left ventricular ejection fraction (LVEF), diameters of cardiac chambers, left ventricular end-systolic and end-diastolic volumes (LVESV; LVEDV), LA area, and valvular hemodynamics,^13^ Experienced cardiologists performed all baseline and follow-up echocardiographic assessments using commercially available ultrasound machines (Vivid S60 and [GE Healthcare, USA] and Philips Affiniti [Koninklijke Philips N.V, the Netherlands]). The database included features of mitral regurgitation, such as the area of the MR jet as a fraction of the left atrial area and vena contracta.

###  Follow-up and outcomes

 Per protocol of the Tehran Heart Center registries, patients were invited for clinic visits and paraclinical assessments at one, six, and 12 months after CABG. TTE was performed at baseline before surgery and at the 12-month follow-up visit. For each patient, the same operator performed TTE at both time points. The outcomes of interest were improved MR based on echocardiographic features and worsening valvular hemodynamics with progression to severe MR. The definition of severe MR was based on the presence of any of the following criteria in TTE: Jet area/LA area ≥ 40%, or vena contracta ≥ 0.7 cm. The study also aimed to investigate the associations of worsening or improvement of mitral valve hemodynamics if there was a sufficient number of patients who experienced these outcomes.

###  Statistical analysis

 Continuous data are presented as mean ± standard deviation. Categorical variables are shown as frequency (percentage). Comparisons were planned between the patients with stable, worsened, and improved MR. Data were examined using IBM SPSS Statistics for Windows, version 23.0 (Armonk, NY: IBM Corp.). A P < 0.05 was considered statistically significant.

## Results

 In this study, we retrieved the data of 291 patients with pre-existing moderate functional MR and included echocardiography data before and after surgery. The mean age of the population was 66.1 ± 9.6 years, and 204 patients (70.1%) were males. The most common cardiovascular risk factors were dyslipidemia (278 patients; 95.9%) and hypertension (251 patients; 86.3%). Most of the study population had extensive CAD, as 240 patients (82.5%) had a three-vessel disease. Moreover, 101 patients (34.7%) had suffered a prior myocardial infarction, and 145 patients (49.8%) had an LVEF ≤ 40% before surgery. Baseline demographic and clinical characteristics and echocardiographic features of the study population are presented in Table 1.

**Table 1:** Baseline characteristics

**Characteristic**	**Total population (n=291)**	**Worsened MR (n=11)**	**Improved MR (n=4)**
Age, years	66.1 ± 9.6	67.7 ± 7.8	55.0 ± 4.8
Male sex	204 (70.1%)	5 (45.5%)	2 (50.0)
Body mass index, kg/m2	26.8 ± 4.3	25.3 ± 4.0	34.1 ± 6.6
Diabetes mellitus	107 (36.8%)	2 (18.2%)	1 (25.0)
Hypertension	251 (86.3%)	9 (81.8%)	1 (25.0)
Smoking	81 (29.2%)	3 (33.3%)	2 (50.0)
Dyslipidemia	278 (95.9%)	10 (90.9%)	3 (75.0)
Family history of CAD	100 (34.4%)	8 (72.7%)	1 (25.0)
Previous myocardial infarction	101 (34.7%)	3 (27.3%)	0 (0)
Systolic blood pressure, mmHg	122.3 ± 9.1	120.9 ± 9.4	115.0 ± 0.5
Diastolic blood pressure, mmHg	90.5 ± 10.9	88.6 ± 7.1	8.2 ± 0.5
Atrial fibrillation	84 (29.3%)	4 (36.4%)	1 (25.0)
Echocardiography			
LVEF, %	42.0 ± 9.9	44.1 ± 10.9	43.7 ± 13.1
LVESV, mL	123.8 ± 38.4	111.7 ± 38.5	99.7 ± 41.8
LVEDV, mL	80.1 ± 32.9	68.3 ± 35.6	52.0 ± 32.5
Mitral regurgitation vena contracta	4.2 ± 0.6	4.0 ± 0.5	3.1 ± 0.3
LA size, mm	38.8 ± 4.9	38.2 ± 2.6	38.0 ± 4.2
LA area, cm2	20.5 ± 4.3	19.5 ± 3.8	21.0 ± 2.1
MR/LA area, %	25.7 ± 11.3	25.1 ± 4.7	16.3 ± 4.4
PAPs, mmHg	33.4 ± 9.3	37.0 ± 13.2	34.7 ± 12.3
Tricuspid regurgitation	227 (78.0%)	9 (81.8)	3 (75.0)
Mild	201 (88.5%)	7 (77.8%)	3 (100.0)
Moderate	26 (11.5%)	2 (22.2%)	0 (0.0)
Mitral annulus dilation			
Mild	3 (1.1%)	0 (0.0)	2 (50.0)
Moderate	272 (97.5%)	11 (100.0%)	2 (50.0)
Severe	4 (1.4%)	0 (0.0)	0 (0.0)
Myocardial segments status			
Apical			
Nl/Mild/hypokinesia	211 (72.5)	9 (81.8)	2 (50.0)
Akinesia	78 (28.6)	2 (18.2)	2 (50.0)
Aneurysmal	2 (0.7)	0 (0)	0 (0)
Lateral			
Nl/Mild/hypokinesia	255 (87.6)	11 (100)	4 (100)
Akinesia	36 (12.4)	0 (0)	0 (0)
Aneurysmal	0 (0)	0 (0)	0 (0)
Anterior			
Nl/Mild/hypokinesia	271 (93.1)	11 (100)	4 (100)
Akinesia	20 (6.9)	0 (0)	4 (100)
Aneurysmal	0 (0)	0 (0)	0 (0)
Inferior			
Nl/Mild/hypokinesia	198 (68.0)	9 (81.8)	4 (100)
Akinesia	89 (30.6)	2 (18.2)	0 (0)
Aneurysmal	4 (1.4)	0 (0)	0 (0)
Septal			
Nl/Mild/hypokinesia	214 (73.5)	7 (63.6)	4 (100)
Akinesia	77 (26.5)	36.4)	0 (0)
Aneurysmal	0 (0)	0 (0)	0 (0)
AntSeptal			
Nl/Mild/hypokinesia	232 (79.7)	9 (81.8)	2 (50.0)
Akinesia	59 (20.3)	2 (18.2)	2 (50.0)
Aneurysmal	0 (0)	0 (0)	0 (0)
Posterior			
Nl/Mild/hypokinesia	227 (78.0)	9 (81.8)	4 (100)
Akinesia	64 (22.0)	2 (18.2)	0 (0)
Aneurysmal	0 (0)	0 (0)	0 (0)
Coronary angiography			
Left main	3 (1.0%)	0 (0.0)	0 (0.0)
SVD	4 (1.4%)	0 (0.0)	0 (0.0)
2VD	44 (15.1%)	2 (18.2%)	1 (25.0)
3VD	240 (82.5%)	9 (81.8%)	3 (75.0)

Abbreviations: CAD: coronary artery disease; LA: left atrium; LVEF: left ventricular ejection fraction; LVEDV: left ventricular end-diastolic volume; LVESV: left ventricular end-systolic volume; MR: mitral regurgitation; PAP: pulmonary artery pressure; SVD: single vessel disease; 2VD: two-vessel disease; 3VD: three-vessel disease.

 The median follow-up duration between surgery and the follow-up TTE was 10.1 months (25^th^-75^th^ percentile: 9.0-11.2). After comparing the baseline and follow-up TTE results, it was demonstrated that only four patients (1.4%) had improvement in MR. The TTE characteristics of patients who had a regression in the degree of MR are shown in Table 2. Due to the low number of patients with improved MR, a pre-specified analysis to determine associations of MR improvement was not feasible.

**Table 2:** Echocardiographic characteristics of patients with improvement mitral regurgitation in follow-up

	**Age and sex**	**Baseline** **LVEF, %**	**Follow-up** **LVEF, %**	**Baseline** **VC, mm**	**Follow-up** **VC, mm**	**Baseline** **LA area, cm**^2^	**Follow-up** **LA area, cm**^2^	**Baseline** **MR/LA area, %**	**Follow-up** **MR/LA area, %**
Patient 1	54 y/o male	30	30	3	< 3	23	23	21.7	17.4
Patient 2	62 y/o female	55	55	3	< 3	22	27	13.6	13
Patient 3	52 y/o male	35	40	3	< 3	21	23	11.9	9.1
Patient 4	52 y/o female	55	50	3.5	< 3	18	16	17.8	19.4

Abbreviations: LA: left atrium; LVEF: left ventricular ejection fraction; MR: mitral regurgitation; VC: vena contracta.

 Eleven patients (3.8%) in the study population showed worsening MR hemodynamics after isolated CABG. In this group of patients, mean LVEF dropped from 44.1 ± 10.9 at baseline to 41.8 ± 11.5 during follow-up. Moreover, the mean LA area increased from 19.5mm ± 3.8mm to 21.7mm ± 3.6mm during the study period. The individual characteristics of patients with worsening MR are shown in Table 3. Table 4 summarizes the echocardiographic features of patients with improvement and worsening of MR, It is of notice that only 4/291 patiens showed MR improvement and 11/291 revealed worsening of MR during follow up.

**Table 3:** Echocardiographic characteristics of patients with worsened mitral regurgitation in follow-up

	**Age and sex**	**Baseline** **LVEF, %**	**Follow-up** **LVEF, %**	**Baseline** **VC, mm**	**Follow-up** **VC, mm**	**Baseline** **LA area, cm**^2^	**Follow-up** **LA area, cm**^2^	**Baseline** **MR/LA area, %**	**Follow-up** **MR/LA area, %**
Patient 1	75 y/o female	50	40	3.3	4	25	26	16	19.2
Patient 2	82 y/o male	50	50	3.5	3.9	23	24	22.6	20.4
Patient 3	66 y/o female	55	45	3.5	5.6	22	27	22.7	25.9
Patient 4	72 y/o male	35	30	3.5	4	12	16	29.3	25
Patient 5	71 y/o female	45	45	4	4.5	22	22	22.7	27.3
Patient 6	54 y/o male	40	30	4	3	18	21	30	23.3
Patient 7	67 y/o female	55	55	4	3.9	18	20	27.8	30
Patient 8	65 y/o male	35	30	4	4	18	19	25	26.3
Patient 9	64 y/o female	45	55	4.5	5	19	19	23.7	23.7
Patient 10	58 y/o male	20	25	4.5	4.5	15	19	33.3	26.3
Patient 11	71 y/o female	55	55	5	5	22	26	22.7	26.9

Abbreviations: LVEF, left ventricular ejection fraction; VC, vena contracta; LA, left atrium; MR, mitral regurgitation.

**Table 4:** Echocardiographic features during follow-up

**Echocardiographic parameter**	**Total population** **(n=291)**	**Worsened MR** **(n=11)**	**Improved MR** **(n=4)**
LVEF, %	44.1 ± 9.8	41.8 ± 11.5	43.8 ± 11.1
LVDd, mm	49.7 ± 7.4	51.8 ± 6.7	50.2 ± 9.1
LVDs, mm	34.3 ± 8.4	37.4 ± 8.3	33.5 ± 8.8
Mitral regurgitation vena contracta, mm	4.3 ± 0.6	4.3 ± 0.7	2.9 ± 0.0
LA area, cm^2^	20.2 ± 3.9	21.7 ± 3.6	22.2 ± 4.6
MR/LA area, %	24.3 ± 5.9	24.9 ± 3.1	14.7 ± 4.6
PAPs, mmHg	27.7 ± 8.0	28.4 ± 12.0	31.2 ± 9.4

Abbreviations: LA: left atrium; LVEDd: left ventricular end-diastolic diameter; LVEDs: left ventricular end-systolic diameter; LVEF: left ventricular ejection fraction; MR: mitral regurgitation; PAP: pulmonary artery pressure.

## Discussion

 In this registry-based study from a tertiary cardiovascular center, we found a surprisingly low rate of 1.4% improvement in MR after isolated CABG surgery among patients with preoperative moderate functional MR. On the other hand, 3.8% of patients experienced a worsening of MR hemodynamics. The low number of patients with either regression or progression of MR meant that conducting further analyses to determine the clinical associations of change in MR severity was not feasible.

 The existing literature about the course of MR after isolated CABG is heterogeneous, partly because the studies have been conducted in diverse clinical settings and partly because of the relatively small sample sizes that were investigated. In the study by Lam et al among 156 patients who underwent isolated CABG with moderate IMR, the degree of MR was variable in the early postoperative period, as 73% of cases had regressed to mild or absent MR, and 6% had progressed to severe MR. However, at six weeks after surgery, MR was mild or absent in 40% and severe in 22% of patients. Notably, the course of MR did not show any association with the extent of CAD or the degree of LV dysfunction.^9^ Another study of 104 patients with functional MR and CAD found that, on average, MR grade was reduced after isolated CABG surgery.^8^ In the study by Mustonen et al 131 patients with ischemic MR were followed up after isolated CABG. The investigators reported that the proportion of patients with mild, moderate, and severe MR at baseline was 66%, 31%, and 3%, respectively, and six years after CABG, 27% had moderate MR, and 6% suffered from severe MR. The study also found that low LVEF was an independent predictor of poor prognosis.^14^

 In functional MR, as a consequence of a diseased and dilated LV, the papillary muscles are displaced, and the resulting leaflet tethering in tandem with annular dilation causes sub-optimal leaflet coaptation.^15^ In this setting, revascularization and restoration of coronary flow in the presence of viable myocardium are expected to result in the recovery of global and regional wall motion and reverse LV remodeling.^16,17^ Moreover, improvement in LV function after revascularization is hypothesized to improve mitral valve function due to reductions in LV dimensions and increased closing forces.^5^ In this framework, improvements in MR after isolated CABG would be expected; however, the observations of the present study, in addition to the previous literature, suggest that the pathophysiology of IMR is much more complex. The rate of improvement in MR after isolated CABG is not considerable. The LV anatomy, papillary muscles, and the mitral annulus profoundly influence ischemia and mitral valve function interactions. Clinical evidence demonstrated that the function of papillary muscles in MR is impaired in terms of both the anatomical configuration and their dynamic perfusion.^5,18^ Moreover, as papillary muscles are located deeply inside the LV, they tolerate a high shear force, which increases the likelihood of complete or partial rupture, elongation, and dyssynchrony. It may be suggested that revascularization and reperfusion only address one side of this pathophysiological complex while failing to restore the balance of all forces inside the LV.

 Previous research has highlighted several associations and predictors of MR regression after coronary revascularization. In an analysis of 6 647 patients with functional MR who underwent percutaneous coronary intervention (PCI), 16% had improved MR 12 months after PCI.^10^ An investigation of patients with varying degrees of MR undergoing isolated CABG revealed that the use of beta-blockers, longer cross-clamp time, and absence of prior CVA as a marker of lower atherosclerotic burden were associated with regression of MR. Moreover, improvements in LV dynamics, in terms of reductions in LVEDV and LVESV, and an increase in LVEF were markers of improved MR during follow-up.^19^ Furthermore, the association of earlier operation timing with improvements in MR has been shown.^20^ In another study of 135 patients with CAD and IMR who underwent isolated CABG, it was shown that a more considerable extent of the viability of the myocardium and a lesser extent of papillary muscle dyssynchrony were associated with MR regression one year after surgery.^21^ This is in keeping with the hypothesis that papillary muscles play a crucial role in the malfunction of the mitral valve in secondary MR. On the other hand, it has been shown that a greater amount of myocardial scar, and the presence of left bundle branch block, as a marker of dyssynchrony in the LV depolarization, is associated with worsening of MR after CABG.^22,23^

 Finally, the evidence regarding the efficacy of mitral valve surgery in patients with mild to moderate IMR who are candidates for CABG is sparse. It should be noted that the latest guidelines recommend concomitant mitral valve surgery with a class of 2a in patients with severe functional MR when CABG is performed to treat CAD;^7^ however, the evidence behind this recommendation comes mainly from non-randomized studies.^24^ Nonetheless, for patients with lesser degrees of MR, there is currently no evidence for performing mitral valve intervention in addition to CABG, despite the progression to severe MR in a subset of patients. The seminal Cardiothoracic Surgical Trials Network (CTSN) trial compared isolated CABG to mitral valve repair plus CABG in patients with moderate MR. This study found that although mitral repair was associated with a more durable correction of MR, it did not result in better LV reverse remodeling, did not reduce adverse events, and did not increase survival.^25^ A 2016 meta-analysis of four randomized controlled trials confirmed no role for concomitant mitral valve surgery in addition to CABG in patients with moderate MR.^4^

 The low number of patients with either regression or progression of MR rendered the pre-specified analysis for identifying clinical associations of improved or worsened MR impractical. First, our study was conducted in a tertiary cardiovascular hospital, which means our population could be inherently different and have more comorbidities than patients in other centers. This hypothesis may explain why so few patients experienced an improvement in MR. Second, a larger sample size with a longer follow-up duration can present a more robust result.

## Conclusion

 In this study from a tertiary cardiovascular center, we found that in patients with preexisting functional MR, isolated CABG did not significantly change the degree of MR after one year of follow-up. Future multi-center studies with larger samples could accurately identify the natural course of function after isolated CABG in this group of patients and determine the clinical associations of MR regression and progression.

## Competing Interests

 None declared.

## Ethical Approval

 All patients participating in the Tehran Heart Center registries provided written informed consent to agree to use their information in anonymized databanks and to be reported in future studies. The institutional review board and the Research Ethics Committee of Faculty of Medicine, Tehran University of Medical Sciences, approved the study protocol (Code: 26862t).
